# A Case of Ectopic Thyroid Presenting as a Superior Mediastinal Mass

**DOI:** 10.7759/cureus.9541

**Published:** 2020-08-03

**Authors:** Takayuki Imai, Yohei Morishita, Shigemi Ito, Satoshi Saijo, Yukinori Asada

**Affiliations:** 1 Head and Neck Surgery, Miyagi Cancer Center, Natori, JPN; 2 Diagnostic Radiology, Miyagi Cancer Center, Natori, JPN; 3 Pathology, Miyagi Cancer Center, Natori, JPN

**Keywords:** thyroid dysgenesis, mediastinum, surgical procedures, diagnostic imaging

## Abstract

We report a very rare case of an ectopic thyroid in the superior mediastinum, which was detected incidentally using imaging. The case was a 50-year-old woman patient. She had an orthotopic thyroid and normal thyroid function. This superior mediastinum mass obviously lacked continuity with the orthotopic thyroid. Its computed tomography density was lower than that of the orthotopic thyroid, and an enhancement was heterogeneously observed. In the cytodiagnosis, only large and small lymphocytes were observed, and malignant diseases such as malignant lymphoma could not be ruled out, so surgical resection was performed through a cervical incision. Combined resection of the thyroid was unnecessary, and ligation of the feeding vessels from the thorax side was able to be carried out without incident.

## Introduction

The most common mediastinal tumors are thymomas, neurogenic tumors, and benign cysts, which account for 60% of all mediastinal tumors [1]. Differential diagnosis is different in children and adults, where neurogenic tumors are more common in children and lymphomas are most common in adults [2]. Although goiters often extend to the substernal mediastinum, primary ectopic thyroid tissues in the mediastinum are very rare entities [3-5]. We experienced a case in which thymoma, malignant lymphoma, and Castleman’s disease could not be ruled out in the differential diagnosis of the superior mediastinal mass, and the diagnosis of ectopic goiter could be confirmed using surgical resection. Because ectopic mediastinal thyroid tissue receives blood flow from the thorax, it has been reported that sternotomy and thoracotomy approaches are essential [6]. However, there have been several reports of mediastinal ectopic thyroids surgically resected via only a cervical approach, all of which involved concomitant resection of the orthotopic thyroid [7-9]. The superior mediastinal mass in this case was clearly separated from the orthotopic thyroid. Here, we present a case of ectopic mediastinal thyroid tissue resected through a cervical incision approach without concomitant resection of the orthotopic thyroid gland.

## Case presentation

A 50-year-old woman was referred to our department with a superior mediastinal mass detected using imaging. She had been receiving outpatient treatment at a local medical clinic for chronic cough for four months. Plain computed tomography (CT) of the lung was carried out because of the poor improvement of her symptoms, but a superior mediastinal mass was incidentally pointed out. Her medical history included cervical carcinoma in situ, but there were no other notable findings. She had no history of allergies or smoking, and alcohol consumption was intermittent.

**Figure 1:**
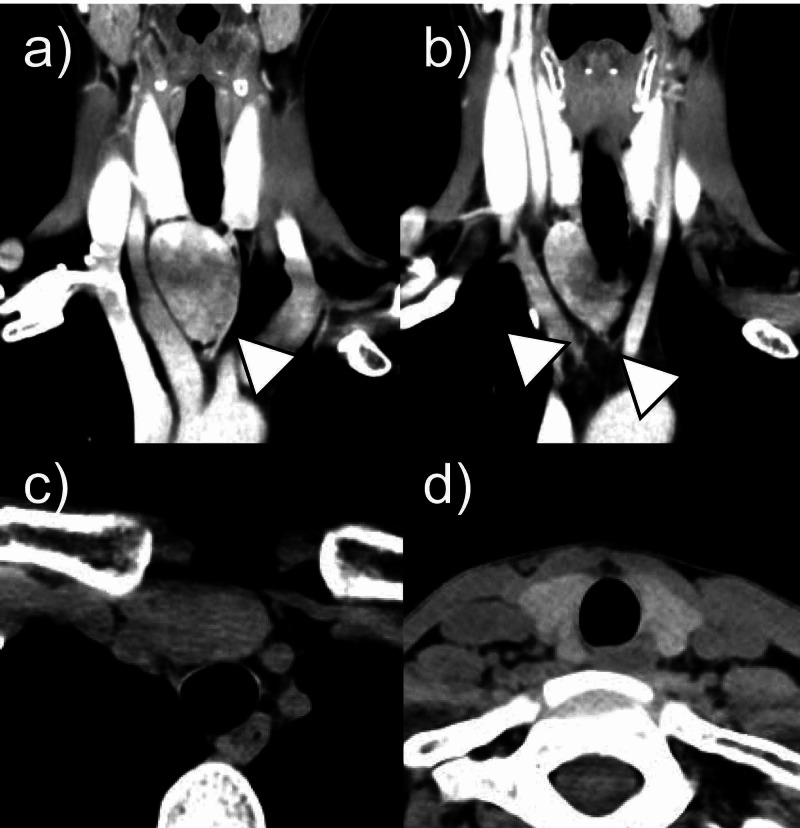
CT findings of the present case. (a, b) Contrast-enhanced CT coronal image. The arrowheads indicate the vasculatures entering the mass from the thoracic sides. (c) Plain CT findings of the mass located in the mediastinum. (d) Plain CT findings of the orthotopic thyroid gland.

Contrast-enhanced CT revealed a well-demarcated mass measuring 40 mm in diameter, extending from the thoracic inlet to the superior mediastinum (Figure 1a, 1b). Orthotopic thyroid tissue was observed, and the mass lesion was not continuous with the thyroid gland. The lesion was slightly less well absorbed than the thyroid tissue (Figure 1c, 1d) and was enhanced somewhat heterogeneously. The vasculature entering the mass from the thoracic sides could be observed (Figure 1a, 1b). Laboratory tests showed normal white blood cell counts and a negative C-reactive protein level. Her serum interleukin-2 receptor level was within the normal range (271.0 U/mL), and serum toxoplasma IgM and IgG antibodies were both negative. Thyroid functions were also within normal limits, and serum-free T4 was 0.99 ng/dL, serum-free T3 was 2.61 pg/mL, and thyroid-stimulating hormone was 1.25 μIU/mL; thereafter, both antithyroid peroxidase antibody and antithyroglobulin antibody levels were normal. Only an elevated serum thyroglobulin level of 87.7 ng/mL could be measured. Ultrasonography also showed clear separation of the mass from the orthotopic normal thyroid, and echo-guided fine needle aspiration cytology specimens showed only large and small lymphocytes.

**Figure 2:**
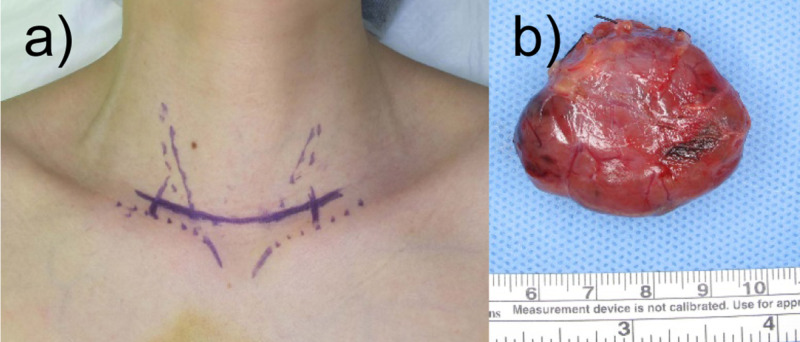
Skin incision lines (a) and excised tissue (b).

Based on these results, thymoma, malignant lymphoma, and Castleman’s disease could not be ruled out, and it was decided to perform surgical resection. We consulted with a respiratory surgeon before the operation, and prepared for sternotomy, although unlikely, when the surgical approach was difficult. Transcervical resection of the superior mediastinal mass was performed (Figure 2a). Because the thyroid gland was clearly separated from the mass, it was preserved, and no combined resection was performed. Multiple inflow vessels from the thorax into the mass were confirmed during surgery. All were carefully ligated, and the mass could be resected through the neck without any issues. The mass seemed to have independent blood supply from those vessels. The recurrent laryngeal nerve could be preserved without needing to be identified. The resected specimen was a mass macroscopically suggestive of thyroid tissue itself (Figure 2b). Operation time was 70 minutes, and blood count was 10 mL. The postoperative course was uneventful and without any complications.

**Figure 3:**
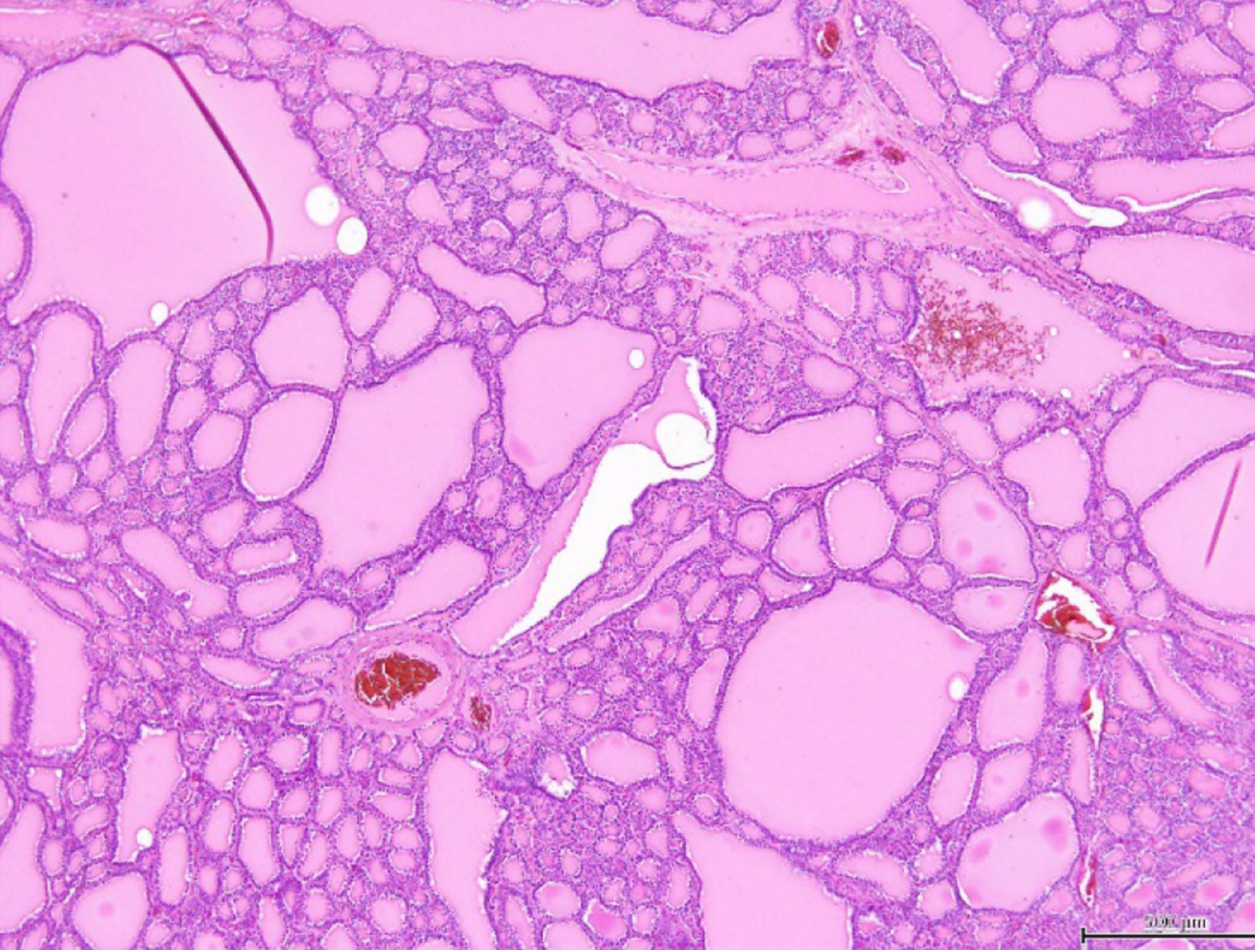
Pathologic finding. Scale bar: 500 μm.

Histopathological examination revealed ectopic thyroid tissue and a nodular goiter consisting of large and small colloid follicles (Figure 3). Postoperative laboratory testing also demonstrated that it was euthyroid. 

## Discussion

Ectopic thyroids occur in one in 100,000-300,000 people [5]. However, the existence of ectopic thyroids was reported to be 10% in an autopsy study, suggesting that there is a discrepancy between the frequency of clinical evidence and actual anatomical presence or remains of ectopic thyroid tissues [10]. It has been reported that 90% of ectopic thyroid glands are lingual thyroids, and ectopic thyroid tissue present in the mediastinum, as in this case, accounts for less than 1% of all ectopic thyroid tissues [4,11]. The incidence of substernal goiters has been reported to range from 0.02% to 0.5%, of which 98% are secondary from the neck and only 1.7% are primary goiters from ectopic thyroids [3]. Mediastinal ectopic thyroid accounts for less than 1% of all mediastinal tumors, and ectopic thyroid tissue in the mediastinum is a very rare occurrence [5].

Ectopic thyroid tissues result from abnormal embryonic development of the thyroid gland [5,6]. In early embryonic life, the thyroid gland, derived from anlage of pharyngeal epithelium, descends from the foramen cecum at the base of the tongue into the neck anterior to the pretracheal region around the second and third tracheal rings, where the thyroid gland is normally located. Thus, as previously described, the majority of ectopic thyroids occur on the descending route of the thyroglossal duct from the base of the tongue [11]. However, the abnormal mechanical relationship between the thyroid gland and the heart during embryonic development has been considered to be involved in the development of ectopic thyroid tissues in the mediastinum, as in this case. It is assumed that as the heart descends during embryonic development, thyroid tissue is pulled caudally to develop an ectopic mediastinal thyroid gland [6].

As for the presence of orthotopic thyroid glands, no eutopic thyroids were observed in 70%-75% of lingual thyroids, and in addition, most of the lingual thyroid cases were hypothyroidism [5,11,12]. In reports of mediastinal ectopic thyroids, they seemed to have orthotopic thyroid and euthyroid function in most of the cases [5,7,8,13-18]. However, Santangelo et al. reported five cases of ectopic thyroids in the mediastinum. One case was a hypothyroidism without any evidence of a eutopic thyroid, and two of the remaining four cases were hyperthyroidism [12]. The present case also had an orthotopic thyroid gland and normal thyroid function, as seen in the majority of cases.

Complaints related to mediastinal ectopic thyroids are often asymptomatic, and those masses were often incidentally found via imaging modalities [9]. Symptoms associated with compression of surrounding tissue by the mass may occur, and cases with dyspnea and cough have been reported in the literature [3,19]. In the present case, the mass slightly compressed the trachea, and the patient suffered from a chronic cough; however, it gradually improved and disappeared at the time of surgery, and was therefore judged to be different from the symptom associated with the mass.

In the present case, cytologic results demonstrated only large and small lymphocytes, so thymoma, Castleman’s disease, and especially malignant lymphoma, which is the most important differential diagnosis in adult mediastinal tumors, could not be ruled out [2]. Furthermore, preoperative diagnosis of ectopic thyroid was not easy because the CT density was lower and the contrast effect was heterogeneous; this is clearly different from the CT findings of orthotopic thyroid tissues. Therefore, we think it would be mandatory to perform surgery in this case. Thyroid scintigraphy may be a candidate diagnostic test for further evaluation. Thyroid scintigraphy, however, has been reported to be of value in patients without an orthotopic thyroid gland or with hypothyroidism but unsuitable for the identification of an ectopic thyroid in other cases [20].

In the present case, the ectopic thyroid in the mediastinum was not connected with the orthotopic thyroid gland, and the blood flow was supplied from the thorax sides, which was demonstrated both on the CT image and in intraoperative findings. It has been reported that sternotomy or thoracotomy is essential for the removal of a mediastinal ectopic goiter, which receives blood flow from the thorax; therefore, in most of the cases, surgical resection was performed through sternotomy or thoracotomy [3,4,6,12,14-17,19]. However, there have been some reports of mediastinum ectopic thyroids resected only through a cervical incision, but in all cases, concomitant resection of the orthotopic thyroids was performed [7-9]. Of course, it is necessary to take appropriate measures depending on the location within the mediastinum. Resection of this ectopic thyroid located in the mediastinum through an external cervical incision was relatively easy because the mass was located at the cranial side. The feeding vessels from the thoracic cavity were ligated without any problem by careful treatment during surgery. Because there was no connection between the ectopic thyroid and the orthotopic thyroid gland, the presence of the orthotopic thyroid gland seemed to have no adverse effect on the surgical field, and therefore combined resection of the thyroid gland was considered unnecessary. We consider that ectopic thyroid in the mediastinum, like in this case, is a disease that should be carefully differentiated for head and neck surgeons because of its rarity and because of fewer reported cases of surgical resection through a cervical incision.

## Conclusions

Ectopic thyroid in the mediastinum is a very rare occurrence. When encountered as a solid tissular mass at the mediastinum, differential diagnosis is very important. Head and neck surgeons should be aware of this rare condition.
